# Supplementary material for: From sequence to enzyme mechanism using multi-label machine learning
Source: BMC Bioinformatics. 2014 May 19;15:150. doi: 10.1186/1471-2105-15-150 (PMC4229970; doi:10.1186/1471-2105-15-150)
Supplement: Additional file 2 — Java code of ml2db. Additional file ml2db_code.tar.gz contains the Java source code to run the multi-label machine learning experiments and save the results to database. The code’s Javadoc is included. [file 1471-2105-15-150-S2.zip › additional file 2/ml2db/ml2db/doc/mulan/classifier/neural/model/NeuralNet.html]

NeuralNet


JavaScript is disabled on your browser.


- Overview
- Package
- Class
- Use
- Tree
- Deprecated
- Index
- Help

- Prev Class
- Next Class

- Frames
- No Frames

- All Classes

- Summary:
- Nested |
- Field |
- Constr |
- Method

- Detail:
- Field |
- Constr |
- Method


mulan.classifier.neural.model

## Interface NeuralNet

- All Known Implementing Classes:
  :   BasicNeuralNet

  ---

    

  ```
  public interface NeuralNet
  ```

  Common interface for interaction with a neural network representation.

  Version:
  :   2012.02.27

  Author:
  :   Jozef Vilcek

- - ### Method Summary

    Methods

    | Modifier and Type | Method and Description |
    | --- | --- |
    | `double[]` | `feedForward(double[] inputPattern)` Propagates the input pattern through the network. |
    | `int` | `getLayersCount()` Returns a total number of layers of the neural network. |
    | `java.util.List<Neuron>` | `getLayerUnits(int layerIndex)` Returns units of a particular layer of the neural network. |
    | `int` | `getNetInputSize()` Gets the size/dimension of the input layer of the neural network. |
    | `int` | `getNetOutputSize()` Gets the size/dimension of the output layer of the neural network. |
    | `double[]` | `getOutput()` Returns the actual output of the neural network, which is a result of last processed input pattern. |
    | `void` | `reset()` Perform reset, re-initialization of neural network. |

- - ### Method Detail


    - #### getNetInputSize

      ```
      int getNetInputSize()
      ```

      Gets the size/dimension of the input layer of the neural network.
      This is the size of input pattern the neural network can process.

      Returns:
      :   the network input size


    - #### getNetOutputSize

      ```
      int getNetOutputSize()
      ```

      Gets the size/dimension of the output layer of the neural network.
      This is the size of output pattern the neural network produces.

      Returns:
      :   the network output size


    - #### getLayersCount

      ```
      int getLayersCount()
      ```

      Returns a total number of layers of the neural network.

      Returns:
      :   the number of layers in the neural network


    - #### getLayerUnits

      ```
      java.util.List<Neuron> getLayerUnits(int layerIndex)
      ```

      Returns units of a particular layer of the neural network.
      The valid indexes for layers are from 0 to N-1, where N is total number of layers
        
      The first layer (index = 0) is always input layer and
      last (index = N-1) always output layer.

      Parameters:
      :   `layerIndex` -

      Returns:
      :   returns an unmodifiable list of units of the particular layer

      Throws:
      :   `java.lang.IndexOutOfBoundsException` - if the index is out of range

      See Also:
      :   `Collections.unmodifiableList(List)`


    - #### feedForward

      ```
      double[] feedForward(double[] inputPattern)
      ```

      Propagates the input pattern through the network.

      Parameters:
      :   `inputPattern` - the input pattern for the network to process

      Returns:
      :   the output of the network

      Throws:
      :   `java.lang.IllegalArgumentException` - if input pattern is null or does not match network input dimension


    - #### getOutput

      ```
      double[] getOutput()
      ```

      Returns the actual output of the neural network,
      which is a result of last processed input pattern.

      Returns:
      :   the output of the network.
          Returns null if network is reset or no input pattern was processed


    - #### reset

      ```
      void reset()
      ```

      Perform reset, re-initialization of neural network.
      All learned knowledge stored in the network will be lost.


- Overview
- Package
- Class
- Use
- Tree
- Deprecated
- Index
- Help

- Prev Class
- Next Class

- Frames
- No Frames

- All Classes

- Summary:
- Nested |
- Field |
- Constr |
- Method

- Detail:
- Field |
- Constr |
- Method
